# Regional difference on rotavirus vaccine coverage in children with diarrhea in Mozambique, before and during COVID-19 pandemic: a cross-sectional analysis

**DOI:** 10.1186/s12879-025-10750-8

**Published:** 2025-03-19

**Authors:** Marta Cassocera, Adilson Fernando Loforte Bauhofer, Assucênio Chissaque, Benilde Munlela, Esperança Guimarães, Telma Isaías, Carolina Conjo, Braiton Maculuve, Sérgio Chicumbe, Nilsa de Deus

**Affiliations:** 1 Instituto Nacional de Saúde, Marracuene district, Maputo Province, Mozambique; 2 Instituto de Higiene e Medicina Tropical, Universidade Nova de Lisboa, Lisboa, Portugal; 3 Programa Alargado de Vacinação, Minister of Health, Maputo City, Mozambique; 4 Departamento de Ciências Biológicas, Universidade Eduardo Mondlane, Maputo, Mozambique

**Keywords:** Rotarix^®^ coverage, Mozambique, COVID-19

## Abstract

**Background:**

Immunization coverage is a global concern for the Immunization Agenda 2030 due to the Coronavirus disease (COVID-19) pandemic. Prior to the pandemic, Mozambique had a positive impact on reducing all-cause diarrhea hospitalization and rotavirus positivity due to vaccination with the monovalent vaccine against rotavirus (Rotarix^®^). We evaluated rotavirus vaccine coverage in Mozambican children with diarrhea in four sentinel sites before and during the COVID-19 pandemic.

**Methods:**

A cross-sectional analysis between January 2016 and April 2023 was performed using the National Diarrhea Surveillance data from four sentinel sites for children under five years old. The cut-off before and during the COVID-19 period was the date of the first COVID-19 case reported in Mozambique on March 22, 2020. Vaccination cards were used to verify rotavirus immunization status. A two-sample test for equality of proportions of rotavirus coverage before and during the COVID-19 pandemic was performed.

**Results:**

During the COVID-19 pandemic, the rotavirus vaccine coverage was 77.3% (133/172), significantly higher than the 68.6% (771/1124) before the pandemic [difference: 8.7% (95% CI: 1.6 to 15.9); p-value = 0.026]. The two sample test for equality of proportions indicates that at the sentinel site in Zambézia province in the center region of the country, the rotavirus vaccine coverage reduced significantly during the pandemic period compared to the pre COVID-19 pandemic period (difference: -28.1%; 95% CI: -47.8 to -8.3; p-value = 0.028).

**Conclusion:**

Despite national level increase of the rotavirus vaccine coverage, during the COVID-19 pandemic, there was a significant reduction in the sentinel site in the center region of the country. Future rotavirus interventions should target areas with lowest rotavirus vaccine coverage, also, rotavirus diarrheal cases and severity should be monitored in those settings to evaluate the interventions impact.

## Background

Rotavirus is one of the pathogens most attributable to moderate-to-severe diarrhea in children under five years in Mozambique [1]. In September 2015, Mozambique introduced the monovalent vaccine against rotavirus (Rotarix^®^, GlaxoSmithKline Biologicals, Rixensart, Belgium) into the national routine immunization program, which reduced rotavirus positivity and the number of all-cause diarrheal hospitalizations between 2014 and 2017 [2], prior to the Coronavirus disease (COVID-19) pandemic.

Between 2017 and 2019, rotavirus vaccine coverage in children with diarrhea and positive for rotavirus infection was 84.4%. At the time, the vaccine effectiveness was 52% in children aged six to 11 months old with at least one dose of the rotavirus vaccine [3]. A modeling study determined that the introduction of the rotavirus vaccine in Mozambique prevented 4628 deaths and averted US$ 3.1 million in healthcare costs, between 2016 and 2020 [4].

The COVID-19 pandemic, disrupted healthcare services provision, posing a negative impact on vaccines coverage globally [5, 6]. Countries neighboring Mozambique such as Malawi and Tanzania, have previously reported reduced immunization coverage during the COVID-19 pandemic [7, 8]. This has increased the number of unvaccinated and under-vaccinated children [9], placing children at risk for vaccine-preventable diseases.

Mozambique may have setbacks on the positive impact achieved with the rotavirus vaccine immunization schedule due to the COVID-19 pandemic; thus, assessing the vaccine coverage before and during the pandemic will provide information on immunization existing gaps and associated factors, which will serve as insights to policy makers promoting catch-up strategies in order to enable equitable access to immunization.

## Methods

A cross-sectional analysis between January 2016 and April 2023 was performed using National Diarrhea Surveillance data from four sentinel sites. The cut-off date for the start of the COVID-19 pandemic period is the date of the first COVID-19 case reported in Mozambique, on March 22, 2020 [10]. Children born before December 2019 were included in the before COVID-19 pandemic period and children born after February 2020 were included in the during COVID-19 pandemic period. Children born between December 2019 and February 2020, were excluded from the analysis to ensure that the date of birth, age of the first and second doses of the rotavirus vaccine would not overlap both, before and during COVID-19 periods.

Included sentinel sites in this analysis are distributed in the three regions of the country and were all the sentinel sites active in the pre and during COVID-19 pandemic periods. Among the four included sentinel sites, in the southern region of the country in Maputo City one tertiary and one quaternary level sentinel site were included, in the center region of the country one tertiary health facility in the Zambézia province was included and in the northern region of the country one quaternary sentinel site was included.

All participants were children aged up to 59 months with diarrhea as the main reason for seeking healthcare. Eligible participants were children at least four months of age, old enough to receive the first and second dose of rotavirus vaccine at the second and third month of age, respectively, according to the immunization schedule of the country [2, 11]. Also, eligible participants were the ones born after June 30, 2015, which were the ones fulfilling the age criteria for vaccine administration vaccine, considering that the vaccine was introduced in September 2015 [2].

Rotavirus vaccine coverage was defined as the proportion of children who completed the rotavirus vaccine according to the national schedule among the eligible children (i.e., were immunized with the first and second doses for rotavirus) [12]. Vaccination cards were used to verify vaccine administration. Children with no evidence of rotavirus vaccine administration were classified as unvaccinated. Children that received a single dose of rotavirus vaccine were classified as partially immunized.

Timeliness for the first and second doses of rotavirus vaccine was defined as immunization at month two and three according to the Mozambique’s immunization program, respectively [2, 11].

Stratified by COVID-19 period’s relative and absolute frequencies for rotavirus vaccine coverage, rotavirus non-vaccinated coverage and the timeliness for doses one and two were computed. The comparison between the proportions difference was made using the two-sample test for equality of proportions.

Cross-tabulation and simple logistic regression models were made to explore the outcome association (rotavirus vaccine coverage) with the independent variables, stratified by COVID-19 pandemic periods. Independent variables were child’s sex, site, HIV status, low birth weight (defined as being born with less than 2500 grams [13]), underweight and stunting status; for the mothers of the included children the following characteristics were recorded: educational level, marital status, and HIV status. Underweight and stunting status were calculated using the R package “anthro” for children aged up to 59 months [14]. P-values less than 5% were considered statistically significant. We used R version 4.4.0 (Vienna, Austria) to conduct the analysis [15].

## Results

During the COVID-19 pandemic period, rotavirus vaccine coverage was higher than the observed before the COVID-19 pandemic [77.3% (133/172) versus 68.6% (771/1124); difference: 8.6% (95% CI: 1.6 to 15.9); p-value = 0.026]. Proportion of children unvaccinated for rotavirus and timeliness for the first and second doses, did not show any statistical difference before and during the COVID-19 pandemic periods (p-value > 0.05, Table 1).

**Table 1:** Immunization status for rotavirus before and during the COVID-19 pandemic in children with diarrhea

Characteristic	Before COVID-19 % (*n*/*N*)	During COVID-19 % (*n*/*N*)	Difference (95% CI)	*p*-value^1^
Rotavirus vaccine coverage	68.6% (771/1124)	77.3% (133/172)	8.7 (1.6 to 15.9)	**0.026**
Unvaccinated for rotavirus	24.4% (274/1124)	20.3% (35/172)	-4.1 (-10.9 to 2.8)	0.290
Timeliness dose 1	80.7% (686/850)	80.3% (110/137)	-0.4 (-8 to 7.2)	1.000
Timeliness dose 2	78.7% (607/771)	78.2% (104/133)	-0.5 (-8.6 to 7.5)	0.981

^1^ Two sample test for equality of proportions

Before the COVID-19 pandemic, sentinel site, child’s mother marital status and stunting were associated with rotavirus vaccine coverage. Compared to the tertiary sentinel site in the southern region of the country, rotavirus vaccine coverage was 0.48 times less likely in the quaternary site in the southern region (Odds ratio (OR): 0.48; 95% CI: 0.3 to 0.74; p-value = 0.001). In the tertiary site in the center region rotavirus vaccine coverage was less likely than in the tertiary site in the southern region (OR: 0.10; 95% CI: 0.07 to 0.16; p-value < 0.001; Table 2).

Before the COVID-19 pandemic rotavirus vaccine coverage was less likely in children from married/co-habiting mothers (OR: 0.52; 95% CI: 0.37 to 0.73; p-value < 0.001) and in children from widow/divorced mothers (OR: 0.34; 95% CI: 0.16 to 0.74; p-value = 0.005), compared to children from single mothers. Stunted children were less likely of having received rotavirus vaccine than non-stunted children (OR: 0.68; 95% CI: 0.48 to 0.96; p-value = 0.027; Table 2).

**Table 2:** Rotavirus vaccine coverage, simple logistic regression models by COVID-19 pandemic periods and time difference

	Before COVID-19			During COVID-19			Difference (95% CI)^3^	*p*-value^3^
Characteristics	% (*n*/*N*)	OR (95% CI)^1^	*p*-value^2^	% (*n*/*N*)	OR (95% CI)^1^	*p*-value^2^		
**Sex**								
Male	70.4% (475/675)	1		78.9% (75/95)	1		8.5 (-0.9 to 18.1)	0.107
Female	65.9% (296/449)	0.81 (0.63 to 1.05)	0.116	75.3% (58/77)	0.81 (0.4 to 1.67)	0.573	9.4 (-1.9 to 20.7)	0.135
**Site**								
Tertiary site in southern region	87.9% (275/313)	1		86.7% (39/45)	1		-1.2 (-13 to 10.6)	1.000
Quaternary site in southern region	77.5% (200/258)	0.48 (0.3 to 0.74)	**0.001**	87.5% (91/104)	1.08 (0.36 to 2.94)	0.889	10 (1.2 to 18.8)	**0.044**
Tertiary site in center region	43.1% (90/209)	0.10 (0.07 to 0.16)	**< 0.001**	15.0% (3/20)	0.03 (0.01 to 0.11)	**< 0.001**	-28.1 (-47.8 to -8.3)	**0.028**
Quaternary site in northern region	59.9% (206/344)	0.21 (0.14 to 0.31)	**< 0.001**	0.0% (0/3)	-		-	
**Mothers’ education level**								
None	65.7% (67/102)	1		57.1% (4/7)	1		-8.6 (-54 to 36.9)	0.961
Primary	71.3% (258/362)	1.30 (0.81 to 2.06)	0.278	73.9% (34/46)	2.13 (0.37 to 11.1)	0.366	2.6 (-12.1 to 17.4)	0.841
Secondary/above	67.7% (441/651)	1.10 (0.7 to 1.69)	0.680	79.8% (95/119)	2.97 (0.55 to 14.3)	0.172	12.1 (3.5 to 20.6)	**0.011**
**Mothers’ marital status**								
Single	79.2% (190/240)	1		83.3% (20/24)	1		4.1 (-13.9 to 22.2)	0.828
Married/Co-habitation	66.4% (557/839)	0.52 (0.37 to 0.73)	**< 0.001**	76.6% (111/145)	0.65 (0.18 to 1.87)	0.464	10.2 (2.2 to 18.2)	**0.020**
Widow/divorced	56.3% (18/32)	0.34 (0.16 to 0.74)	**0.005**	66.7% (2/3)	0.40 (0.03 to 9.81)	0.495	10.5 (-56 to 76.9)	1.000
**Mothers’ HIV status**								
Negative	70.7% (458/648)	1		78.2% (111/142)	1		7.5 (-0.6 to 15.6)	0.090
Positive	77.1% (192/249)	1.40 (1 to 1.98)	0.054	76.0% (19/25)	0.88 (0.34 to 2.6)	0.810	-1.1 (-19.8 to 17.5)	1.000
**Child HIV status**								
Negative	74.0% (580/784)	1		76.7% (115/150)	1		2.7 (-5.1 to 10.5)	0.556
Positive	71.1% (54/76)	0.86 (0.52 to 1.48)	0.580	80.0% (8/10)	1.22 (0.29 to 8.31)	0.809	8.9 (-23.5 to 41.4)	0.827
**Low birth weight (<2500 g)**								
No	71.2% (607/853)	1		78.6% (110/140)	1		7.4 (-0.5 to 15.3)	0.087
Yes	69.0% (89/129)	0.90 (0.61 to 1.36)	0.614	95.5% (21/22)	5.73 (1.12 to 105)	0.095	26.5 (12 to 40.9)	**0.020**
**Underweight (Z-score < -2)**								
No	69.4% (547/788)	1		78.4% (105/134)	1		9 (0.8 to 17.1)	**0.045**
Yes	66.3% (195/294)	0.87 (0.65 to 1.16)	0.330	87.5% (14/16)	1.93 (0.5 to 12.8)	0.401	21.2 (0.8 to 41.6)	0.137
**Stunting (Z-score < -2)**								
No	72.9% (323/443)	1		79.7% (47/59)	1		6.8 (-5.3 to 18.8)	0.343
Yes	64.6% (148/229)	0.68 (0.48 to 0.96)	**0.027**	76.9% (10/13)	0.85 (0.22 to 4.23)	0.826	12.3 (-15.5 to 40.1)	0.544

^1^ Odds ratio and 95% confidence intervals; ^2^ P-value from simple logistic regression model; ^3^ Two sample test for equality of proportions

During the COVID-19 pandemic, sentinel site was the only factor associated with reductions in rotavirus vaccine coverage. Rotavirus vaccine coverage was less likely in children from the tertiary site in the center region compared to the tertiary site in the southern region (OR: 0.03; 95% CI: 0.01 to 0.11; p-value < 0.001; Table 2).

Two sample difference between the two COVID-19 periods indicates that the tertiary sentinel site in the center region was the only characteristic with a significant reduction of rotavirus vaccine coverage during the COVID-19 period compared to the before COVID-19 period (difference: -28.1%; 95% CI: -47.8 to -8.3; p-value = 0.028). Statistical significant incrementation of the rotavirus vaccine coverage during the COVID-19 period compared to the prior COVID-19 period were observed in the quaternary site in the southern region (difference: 10%; 95% CI: 1.2 to 18.8; p-value = 0.044), in children from mothers with secondary/above educational level (difference: 12.1%; 95% CI: 3.5 to 20.6; p-value = 0.011), in children from married or co-habiting mothers (difference: 10.2%; 95% CI: 2.2 to 18.2; p-value = 0.020), in children born with low-birth (difference: 26.5%; 95% CI: 12 to 40.9; p-value = 0.020) and in children non-underweighted (difference: 9%; 95% CI: 0.8 to 17.1; p-value = 0.045; Table 2).

## Discussion

The global rotavirus vaccine coverage for 2022 was 51% [16], which is lower than that observed in Mozambique before and during the COVID-19 pandemic. Some countries neighboring Mozambique had differences in rotavirus vaccine coverage before and during the COVID-19 pandemic. An increased rotavirus vaccine coverage was observed between the pandemic periods for South Africa (72–83%) and Malawi (75–89%), opposed to those, reduced rotavirus vaccine coverages were observed in Zimbabwe (83–76%), Zambia (87–68%), and Tanzania (89–75%) [17].

The World Health Organization estimates for rotavirus vaccine coverage in Mozambique showed an increased coverage from 67 to 75%, before and during the COVID-19 pandemic [17], which were similar to our sampled estimates.

Both our results and the World Health Organization estimates for rotavirus vaccine coverage in Mozambique indicates that the country failed to achieve 90% target coverage at the national level by 2020 [18]. With the upcoming of the COVID-19 pandemic, the Immunization Agenda 2030 was set with the goal of leaving no one behind, by implementing strategies to limiting disadvantaged vaccine coverage, such as geographical differences, and promoting evidence-based approaches to identify and eliminate vaccination barriers [19].

Throughout our sentinel sites, the one located in Zambézia province, in the center region of the country, had the lowest rotavirus vaccine coverage and was significantly lower during the COVID-19 pandemic. A previous survey conducted in the same province identified a long waiting period for the vaccine uptake, limited health workers in health facilities on the vaccination date, and no vaccine given to the child due to sickness as barriers for the vaccine uptake [20]. During the COVID-19 pandemic, a qualitative assessment recorded side effects to the routine immunization. The assessment indicated that non-compliance with vaccine uptake was associated with vaccines stockouts and the implementation of new non-pharmacological policies to mitigate the COVID-19 pandemic such as mandatory use of face masks. Also, the health workers reported reduced access to vaccines due to boarders’ closures [21].

Reduced vaccine coverage in the tertiary site in the center region sets back positive impact observed after the rotavirus vaccine introduction in Mozambique. This can be translated into increased susceptibility to severe rotavirus infection in children [2–4, 22], and consequently the hospitalization rate by diarrhea in the pediatric group and circulation of rotavirus in the community. Thus, COVID-19 impact on diarrhea burden in the pediatric group should be assessed.

Before the COVID-19 period, rotavirus vaccine coverage was higher in children from single mothers compared to children from married/co-habiting or children from widow/divorced mother, suggesting that shared decision-making may have had a negative effect on vaccine uptake before the COVID-19 pandemic period.

Within the sentinel sites in the southern region of Mozambique in the pre-pandemic period, rotavirus vaccine coverage was lower in the quaternary site compared to the tertiary site, which could have been influenced by the disease severity. It’s has been observed that the clinical presentation of rotavirus infection is more severe in unvaccinated than in vaccinated children [23], as such, severe cases are more likely to be admitted in the quaternary health facility due to their ability to provide a more specialized treatment than tertiary health facilities [24], for example, controlling the potential effect that comorbidities would have in increasing the disease severity.

Data included in this analysis may be an underestimate of the true vaccine coverage since many children may not have been brought in to seek care for diarrhea symptoms during the COVID-19 period, however, our overall coverage estimates are similar to the ones reported by the WHO [17].

Our data collection sites are distributed in three regions of the country; however, they represent < 1% of all health facilities [25]. Although this poses a limitation on the country’s representativeness, we were able to detect geographic differences within the analyzed sites, which reinforces the need to strengthen the rotavirus immunization coverage in the central region after a baseline assessment is conducted which should include, vaccine availability, acceptability and associated factors. Also, continuous surveillance of rotavirus severity should be assessed in areas immunized and compared to under-immunized areas.

## Conclusions

Despite national level increase of the rotavirus vaccine coverage during the COVID-19 pandemic in children seeking healthcare due to diarrhea at selected sentinel sites in Mozambique, there were significant reductions in the tertiary sentinel site located in the center region of the country. We also observed that child’s mother marital status and stunting were only associated with rotavirus vaccine coverage during the pre-pandemic period. Interventions to strengthen rotavirus immunization in areas with low rotavirus vaccine coverage should be prioritized, by identifying, monitoring and resolving factors associated with lower performance.

## Data Availability

Data used for this analysis can be requested from the Instituto Nacional de Saúde, Mozambique, through the corresponding author. Researchers interested in secondary analysis must submit a research proposal for consideration by the study investigators as well as by the Directorate for Research in Health and Well-Being. Upon approval, the requestor must sign a data use agreement.

## Abbreviations

CI: Confidence interval
COVID-19: Coronavirus disease
HIV: Human Immunodeficiency Virus
OR: Odds Ratio
WHO: World Health Organization
